# Concise Historic Overview of Strain Sensors Used in the Monitoring of Civil Structures: The First One Hundred Years

**DOI:** 10.3390/s22062397

**Published:** 2022-03-20

**Authors:** Branko Glisic

**Affiliations:** Department of Civil and Environmental Engineering, Princeton University, Princeton, NJ 08544, USA; bglisic@princeton.edu; Tel.: +1-609-258-8278

**Keywords:** strain sensors overview, strain-based structural health monitoring, strain gauge, vibrating wire sensors, fiber optic sensors, discrete and 1D distributed sensing, 2D and 3D sensing, sensing paints, skins, sheets, and surfaces

## Abstract

Strain is one of the most frequently monitored parameters in civil structural health monitoring (SHM) applications, and strain-based approaches were among the first to be explored and applied in SHM. There are multiple reasons why strain plays such an important role in SHM: strain is directly related to stress and deflection, which reflect structural performance, safety, and serviceability. Strain field anomalies are frequently indicators of unusual structural behaviors (e.g., damage or deterioration). Hence, the earliest concepts of strain sensing were explored in the mid-XIX century, the first effective strain sensor appeared in 1919, and the first onsite applications followed in the 1920′s. Today, one hundred years after the first developments, two generations of strain sensors, based on electrical and fiber-optic principles, firmly reached market maturity and established themselves as reliable tools applied in strain-based SHM. Along with sensor developments, the application methods evolved: the first generation of discrete sensors featured a short gauge length and provided a basis for local material monitoring; the second generation greatly extended the applicability and effectiveness of strain-based SHM by providing long gauge and one-dimensional (1D) distributed sensing, thus enabling global structural and integrity monitoring. Current research focuses on a third generation of strain sensors for two-dimensional (2D) distributed and quasi-distributed sensing, based on new advanced technologies. On the occasion of strain sensing centenary, and as an homage to all researchers, practitioners, and educators who contributed to strain-based SHM, this paper presents an overview of the first one hundred years of strain sensing technological progress, with the objective to identify relevant transformative milestones and indicate possible future research directions.

## 1. Introduction

Modern design and construction of civil structures are based on safety and serviceability criteria prescribed in practice codes. These criteria involve allowable stresses and deflections, which, in the design phase, are typically predicted using structural analysis. If the stress at a point or stress derivatives (e.g., internal forces: normal force, shear force, and bending moment) in a structural member approach or exceed the limit value (e.g., stress approaches or exceeds the strength of the construction material or the compressive normal force approaches or exceeds the buckling capacity), the integrity of the structure can be imperiled, and its safety compromised. If the deflection of a structural member approaches or exceeds the serviceability limit, the intended function of structure might be impaired. Consequently, assessing stresses and deflections in civil structures represents important objectives of Structural Health Monitoring (SHM). Exceedance of the safety and/or serviceability limits typically reflects the existence of unusual structural behaviors (e.g., damage or deterioration), and thus an additional important objective of SHM is the characterization of these unusual structural behaviors (i.e., their detection, localization, and quantification). While these three objectives are challenging to achieve in real-life settings, strain-based SHM can greatly help meet these challenges.

**Figure 1:**
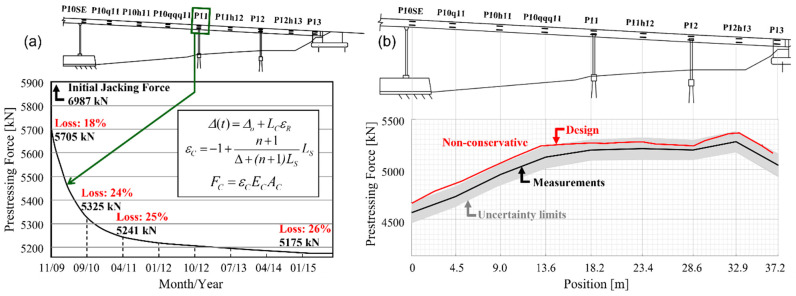
Stress-related parameters (stress derivatives) inferred from long-term strain-based SHM of a real structure (Streicker Bridge): (**a**) loss of prestressing force over several years, and (**b**) distribution of prestressing force along the full length of the bridge and its comparison with design values (modified from the slides of the author’s university course CEE 537 Structural Health Monitoring).

The first challenge lies in the fact that generally applicable sensors for direct monitoring of stress in real-life settings do not exist. While there are exceptions, they are rather limited to very specific applications and cannot be generalized: for example, an elastomagnetic stress sensor can be applied for monitoring tension in cables, e.g., [1,2] but cannot be applied to steel beams or concrete structures. Strain is a parameter that is in direct relation with the stress through constitutive equations, and any change in the latter would result in a change in the former. Strain sensors can be deployed for virtually any type of structure and any construction material; thus, if the constitutive equations of the construction material are known, strain sensors can be applied to determine the stress or stress derivatives from strain, as witnessed by many real-life examples (e.g., [3,4,5,6,7,8,9,10,11]). As an illustration, Figure 1a shows the loss of prestress force over several years that was inferred through strain-based SHM at one cross-section of a real bridge (Streicker Bridge, Princeton, NJ, USA), and Figure 1b shows the distribution of prestress force along the bridge and its comparison with design values [12].

**Figure 2:**
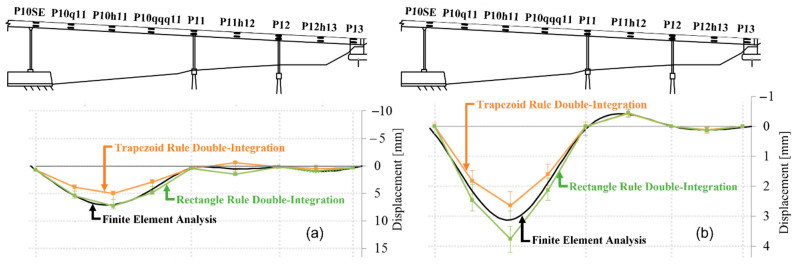
Deformed shapes (deflections) inferred from strain-based SHM (using trapezoid and rectangular rules for double integration of curvature) of a real structure (Streicker Bridge) and their comparison with numerical models: (**a**) due to removal of formworks during construction and (**b**) during load test of the bridge (modified from the slides of author’s university course CEE 537 Structural Health Monitoring).

As opposed to stresses, deflections can be directly monitored in real-life settings using a variety of contact or non-contact sensors. Examples of the former are linear variable differential transformers—LVDTs and hydrostatic systems (e.g., [13,14]), and examples of the latter are cameras (e.g., [15,16]), global positioning system—GPS (e.g., [17,18]), laser-based sensors (e.g., [19,20]), and radar-based sensors (e.g., [21]). However, even though these sensors enable direct monitoring of deflections, their implementation in real-life settings faces significant challenges that render them impractical for long-term monitoring: they require stable reference points, an unobstructed line of sight, and clear targets (e.g., [22,23]). However, strain sensors do not feature such limitations, and if a sensor network is properly designed it can be used to indirectly monitor deformed shapes, i.e., the principal constituent of deflection. For example, in the case of beam-like structural members, the curvature distribution can be inferred from strain measurements, followed by determination of deformed shapes by using double-integration of the curvature (e.g., [24]). As an illustration, Figure 2 shows deformed shapes inferred through strain-based SHM of a real bridge (Streicker Bridge) due to form removal and load testing [24].

**Figure 3:**
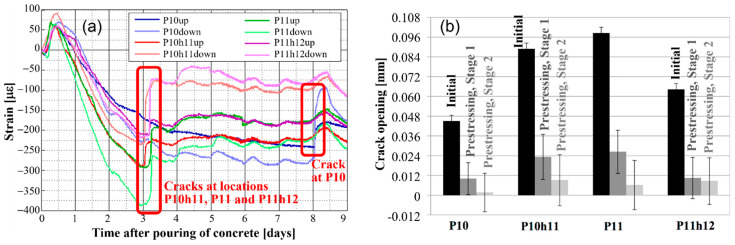
Damage detection and characterization using a strain-based SHM for a real structure (Streicker Bridge): (**a**) “jumps” in strain time series enable damage detection (crack occurrence) and quantification, (**b**) evaluation of residual crack size after two prestressing stages enables assessment of structural condition and performance (modified from the slides of author’s university course CEE 537 Structural Health Monitoring).

Finally, identification (detection, localization, quantification, and prognosis) of unusual structural behaviors (e.g., damage or deterioration) is particularly challenging in real-life settings due to variability of loads and environmental influences, and its success relies on the selection of appropriate damage-sensitive features. The first signs of unusual behavior frequently manifest in the form of strain-field anomalies (e.g., cracking, bowing, excessive rheological strain, differential settlement of foundations, reduction in cross-section due to corrosion, spalling or alkali-silica reactions, etc.). Consequently, strain was often selected as a damage-sensitive feature (e.g., [25,26,27,28]), as well as myriad of strain derivatives such as (but not limited to) the position of the neutral axis [28], curvature [27], prestressing force (e.g., [12]), cross-sectional stiffness (e.g., [28]), cross-sectional integrity (e.g., [27,28]), and thermal “signatures” (e.g., [29]). An important reason for strain-based monitoring is the capacity to not only characterize unusual structural behaviors (i.e., detect them, localize them, and quantify them), but also to make prediction on current and future conditions of a structure (e.g., [12,26,27,28]), i.e., to achieve the so-called Level IV SHM [30]. As an illustration, Figure 3 shows the strain-based detection of thermal cracking at multiple locations of a bridge (Streicker Bridge) and evaluation of cracking closure after prestressing [31].

The examples shown above, while not comprehensive, clearly illustrate the currently achieved “power” of strain-based SHM. While they all include application for a bridge structure, strain-based SHM demonstrated similar success when applied to virtually any other type of civil structure and infrastructure: high-rise buildings (e.g., [26,32]), tunnels (e.g., [11,33]), pipelines (e.g., [34,35]), dams [11,36], wind turbines (e.g., [37]), geotechnical works such as shafts and insets, foundations, and retaining walls (e.g., [6,11,25]), etc.

The journey to realize these achievements was practically 100 years long and was built on collaborative and cumulative interdisciplinary efforts of numerous researchers, educators, and practitioners. The importance of measuring and monitoring the strain in civil structures was identified in the XIX century. Developments in electrical engineering in the first half of the XX century led to the invention of the first strain sensors. These sensors were discrete (point sensors), based on electrical principles, and mostly featured a short gauge length. In this paper, they will referred to as the first-generation sensors. In the second half of the XX century, developments of fiber-optic technologies led to breakthroughs in strain sensing: in addition to discrete short-gauge fiber optic strain sensors (FOSS), discrete long-gauge and truly distributed one-dimensional (1D) fiber-optic strain sensors were invented, leading to several paradigm changes in strain-based SHM. While FOSS were transformative, electrical sensors did not lose importance—on the contrary, they were empowered by wireless capabilities, and they retained a significant place in strain-based SHM. In this paper, they will referred to as the second-generation sensors. Finally, in the early XXI century, a variety of new technologies and materials appeared in various areas of engineering, leading to research that aims at creating quasi-distributed and truly distributed two-dimensional (2D), and recently, three-dimensional (3D) strain sensors specifically targeting characterization (detection, localization, and quantification) of unusual structural behaviors. In this paper, they will referred to as the third-generation sensors.

The aim of this paper is to present and analyze the historic development of strain sensors implemented in civil structural health monitoring and their impact on strain-based SHM, so that important milestones can be emphasized and understood and future research directions identified. To keep the paper concise, the focus is mostly on technologies that are either commercially available (for the first and second generations of strain sensors) or at least reached the level of field testing (for the third generation of strain sensors). More details and extensive overviews the on each specific technology can be found in relevant literature, which is referenced in the next sections, where appropriate.

## 2. First Generation: Discrete Short-Gauge Electrical Strain Sensors

**Figure 4:**
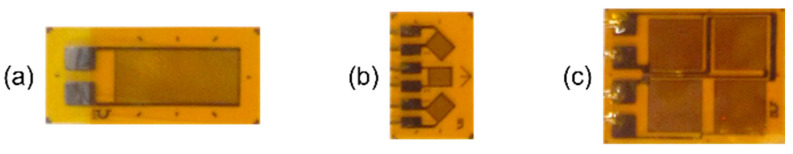
Examples of modern strain gauges with different configurations: (**a**) ordinary strain gauge, (**b**) “rosette”—system of three strain gauges that can infer a 2D strain tensor, and (**c**) full-bridge strain gauge—system consisting of four resistors that specifically use differential measurement to perform thermal self-compensation (modified from the slides of author’s university course CEE 537 Structural Health Monitoring).

Lord Kelvin’s (William Thomson’s) demonstration to the Royal Society of London in 1856 (Bakerian Lecture “On the Electro-dynamic Qualities of Metals”), that electrical resistance in metallic conductors changes when the latter is exposed to mechanical strain [32,33], sparked the first ideas about strain sensors, more precisely about resistive strain gauge. The idea was not immediately implementable at that time, as there were no bonding agents available to enable the installation of a metallic conductor (sensor) in the structure. Another 82 years passed until the resistive strain gauge was de-facto invented in 1938, by Edward E. Simmons and Arthur C. Ruge, independently from each other [38,39]. Strain-gauge, being bondable to a surface, made an invaluable impact in monitoring civil structures, as it enabled simple application to large variety of structural materials such as metal, timber, existing concrete, and composite materials. Simple physical principle enabled a large variety of sensor gauge lengths and configurations; some examples are shown in Figure 4.

**Figure 5:**
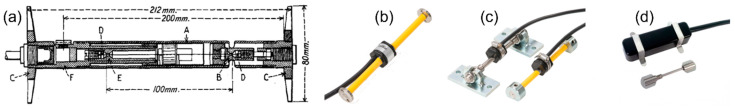
(**a**) Schematic representation of an embeddable VW strain sensor designed by Davidenkoff (reprinted with permission from Ref. [3]. 1934. ASTM International) and examples of modern VW strain sensors: (**b**) embeddable, (**c**) two types of surface mounting, and (**d**) different packagings of surface mounting (top) and embeddable (bottom) VW strain sensors (photos courtesy of Roctest, Saint-Lambert, QC, Canada, www.roctest.com, and Telemac, Gretz-Armanvilliers, France, www.telemac.fr, last accessed on 28 February 2022); sensor sizes in the figure are not to scale.

In the meantime, 19 years before the Simmons and Ruge discoveries, the first strain sensor was invented by German researcher Otto Schaefer in 1919. He did not base his sensor on the principle of change in resistivity in a metallic conductor, but rather on the change in the vibrating frequency of tensioned wire due to a change in its strain. Hence, he translated the principle of a vibrating wire (VW) into a VW strain sensor [40]. Professor N. Davidenkoff, from the Soviet Union, learned about Schaefer’s invention in 1926. He re-created the VW sensor (which he called a “teletensometer”) and made it embeddable in concrete, see Figure 5. After successful testing in the lab, he deployed it in the Zoragetstroi Tunnel in 1931 [3].

At the same time, in France, André Coyne patented his own version of the VW strain sensor in 1931 (he called it “témoin sonore”—sonic witness/sensor) and deployed it in a dam on the La Bromme River [41]. Coyne funded the company Télémac (abbreviation of “Télémesures Acoustiques”—acoustic measures at distance), which, since then, has been manufacturing and applying large variety of sensors based on the VW principle, including strain sensors [42]. Research on VW sensors continued, and, as an example, F. Potocki proposed his own version of an embeddable VW strain sensor in 1958, designed for long-term monitoring of concrete structures [43]. Today, VW strain sensors are widely accepted and applied in numerous monitoring projects worldwide. Photographs of examples of modern VW sensors are given in Figure 5.

**Figure 6:**
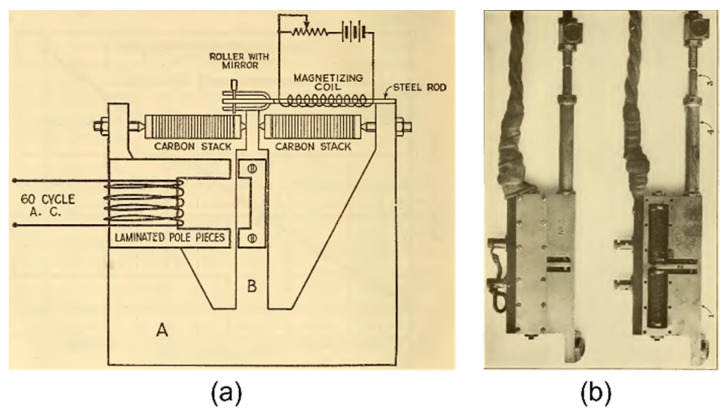
(**a**) Schematic of an electrical telemeter and (**b**) view of closed and open packaging of an electrical telemeter [44].

**Figure 7:**
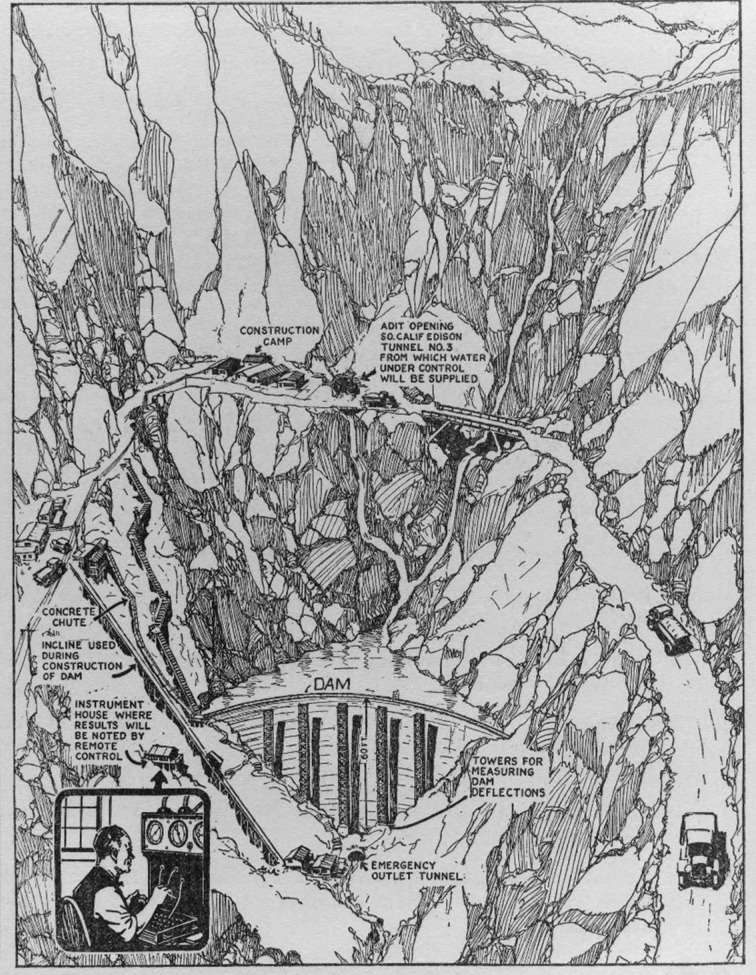
Schematic of the implementation of a monitoring system in Stevenson Creek Experimental Dam (image credit: The Stevenson Creek test dam (1925). Southern California Edison Collection of Photographs (photCL SCE), The Huntington Library, https://go.exlibris.link/461Zn0Q0, last accessed on 28 February 2022).

In parallel with the development of embeddable VW sensors in Europe and the Soviet Union, Burton McCullom and O.S. Peters from the U.S. Bureau of Standards (today the National Institute of Standards and Technology—NIST) conceived, in 1924, a resistive strain sensor consisting of a stack of carbon discs that would change their electrical resistance when subjected to a change in compression. A view of their “electrical telemeter” is given in Figure 6 [44]. These sensors were used in Stevenson Creek Experimental Dam tests (near Fresno, California, USA) 1925–1927 [36]. A schematic of the Stevenson Creek Experimental Dam test is shown in Figure 7. This is one of the first known schematics of the implementation of a remote monitoring system in a civil structure, which was used for dissemination.

**Figure 8:**
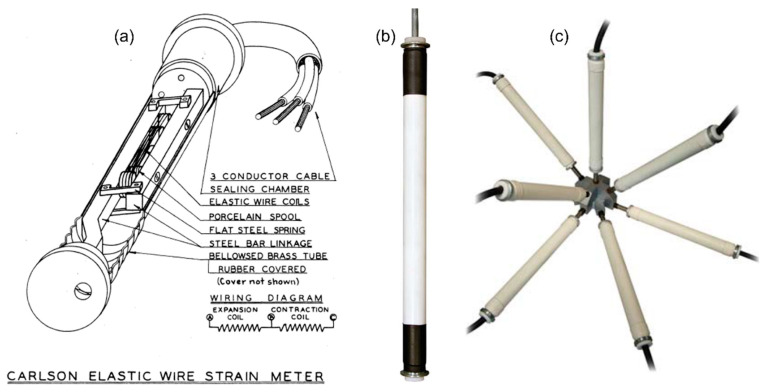
(**a**) View of the components of the Carlson strain meter ( Reprinted with permission from Ref. [45]. 1939. Massachusetts Institute of Technology – MIT), (**b**) modern packaging of the sensor, and (**c**) so-called “spider configuration” with multiple sensors enabling assessment of a 3D strain tensor (photos courtesy of: RST Instruments, Ltd., Maple Ridge, BC, Canada, www.rstinstruments.com, last accessed on 28 February 2022); sensor sizes in the figure are not to scale.

Electrical telemeters suffered from long-time instability and were affected by humidity. Looking for the improvements, in the 1930′s, Roy Carlson developed an embeddable resistive strain sensor (he named it a “strain meter”) that used unbonded carbon steel wire. Two coils of the wire were installed on steel rods connected to anchors (at each end of the sensor), so that when the distance between the anchors was subjected to change, one coil increased in length and the other decreased. The total resistance of the two coils was used to measure temperature, while the ratio in resistance was used to measure strain. The sensor packaging was filled with oil to prevent the penetration of humidity. The sensors were commercialized by Carlson himself, they have shown excellent long-term stability, and have been applied in numerous civil and geotechnical structures, including Hoover Dam [36]. A view of the sensor components is shown in Figure 8 [45], along with photographs of modern appearances of sensors and their configurations.

In addition to VW strain sensors, the Carlson strain meter, and resistive strain gauges, other physical principles were researched for strain sensors. Strain sensors based on the piezo-electric effect were developed (e.g., [46,47]). However, they featured two shortcomings that limited their application in strain-based SHM in civil structures: difficulty in measuring the true static value and sensitivity to transverse strain. While their application in strain-based SHM was not fruitful, sensors with piezo-electric sensing elements found widespread applications in other SHM approaches, such as acoustic and wave-propagation-based SHM (e.g., [48,49]). Given their excellent potential for accurate strain measurement, the research on this type of sensor continues (e.g., [50,51]).

Over the decades, the first-generation electrical strain sensors became an established tool for SHM purposes. Their impact is immeasurable: they opened the doors to understanding the true structural behaviors in real-life settings and of long-term behaviors of construction materials and practically gave birth, not only to strain-based SHM, but also to SHM in general, as well as sparked an accelerated development of SHM through the second half of the XX century. Their development resulted in the creation of numerous companies that represent important industry supporting civil engineering applications. Examples of modern versions of these sensors are given in Figure 4, Figure 5 and Figure 8. The first-generation sensors continue to be extensively used in the XXI century, empowered by wireless sensing capabilities, of which developments started in the last third of the XX century (e.g., [52,53,54]) and rapidly matured in terms of commercialization and field applications (e.g., [32,55,56]). These wireless technologies are mostly based on so-called wireless nodes, which require an energy source, typically provided by a battery or an energy harvesting device. Given that energy sources in some applications can be scarce, a new wave of wireless sensing techniques emerged and rapidly reached commercial maturity in the second decade of the XXI century. These techniques are based on passive radiofrequency technology, which is used to both wirelessly power and read the strain sensor (e.g., [57,58,59,60]).

While each specific type of the first-generation sensor has different advantages and limitations, depending on their physical principle and components, the first-generation strain sensors have some common advantages and limitations, as listed below. Their best performances are given in Table 1. Note that, often, these performances cannot be achieved simultaneously.

General advantages:

- Long tradition: continued improvement in performance and extensive experience in applications make the first-generation strain sensors easy to understand, apply, and operate, which conveys confidence when using them.
- Affordable cost: due to a simple functioning principle requiring inexpensive sensor parts and massive production resulting from their widespread use, the cost of the first-generation sensors is relatively low.
- Excellent measurement performance: the first-generation strain sensors feature high resolution, typically in the range of 1 με (1 με = 1 microstrain = 1 μm/m = 1 × 10^−6^ m/m), and precision typically ranges between 0.5% and 2% of the sensors’ full scale; these measurement performances are suitable for the SHM of civil structures.
- Easy repair of cable extensions: being made of simple electrical wires, the cable extensions, which are carriers of energy and information for the first-generation sensors, are easy to repair and replace onsite in the case of damage.
- Wireless capability: being governed by electrical principles, the first-generation sensors are simple to equip with wireless capabilities, which in turn enable remote and decentralized reading, processing, analysis, and communication of data from sensors and even from the entire sensor network.

General challenges:

- Electromagnetic interference (EMI): being governed by electrical principles, the functioning of first-generation strain sensors might be obstructed by interference from various sources of electromagnetic fields found in the proximity of monitored structures (e.g., electrical power lines and conductors, lightning and thunders, radio waves, etc.); the EMI can alter the measurements, and sometimes result in permanent sensor malfunction or damage; in some cases, mitigation measures can be taken (e.g., EMI shielding), but they incur additional material and installation cost;
- Temperature compensation: except for the Carlson strain meter, sensing elements of the first-generation sensors are affected by environmental temperature changes and consequently, their strain measurements must be thermally compensated, which incurs additional material and installation cost and lowers the accuracy of strain measurement.

More details regarding the history and performance of the first-generation sensors and supporting wireless technologies can be found in relevant literature (e.g., [8,39,42,61,62,63]).

## 3. Second Generation: Discrete Short-Gauge, Long-Gauge, and Distributed Fiber-Optic Strain Sensors

An important limitation in the applicability of the first-generation strain sensors is set by their predominantly short gauge length (see Table 1): it decreases their accuracy in inhomogeneous materials (e.g., concrete [8,11]), thus restricting their use to mostly local material monitoring, and it reduces their ability to detect damage [11]. This is described in more detail as follows.

In civil engineering, the design of structures mostly considers material behavior at a macro scale, regardless of the material true nature. For example, concrete is considered homogeneous at a macro scale, although it is inhomogeneous at a meso scale due to the presence of inclusions (e.g., air pockets and aggregate) and discontinuities (e.g., cracks in reinforced concrete, which are not considered damage). Thus, if monitoring is performed at a structural scale, a strain measurement is expected to provide information on material behavior at a macro scale. To illustrate this statement, let us observe a 5-m-tall prismatic column of concrete with a square cross-section 1 m × 1 m (the area of the cross-section is 1 m^2^). To simplify the illustration, let us assume a vertical load of 300 kN at the centroid of the top cross-section of the column and a modulus of elasticity of concrete of 30 GPa. Then, at any point of the cross-section in the middle of the column, the theoretical estimate of the strain due to the load only is ε = 3 × 10^5^/(30 × 10^9^ × 1) = 10^−5^ = 10 με. Note that, theoretically, this value is constant across the observed cross-section. However, the true value of the strain at any point of the observed cross-section will depend on the exact material properties at that point. For example, at some points, the material will be cement paste, at other points it will be an aggregate, and at others it might be a discontinuity: air pocket, or pore, with or without water. Hence, within the same cross-section, under a described load, the true strain will be variable and not constant. However, the average value of the strain across the cross-section will be equal to theoretical estimate of 10 με.

**Figure 9:**
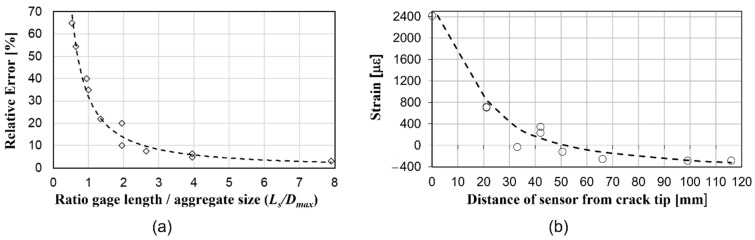
(**a**) Example of experimental results of dependence of error in strain measurement in concrete as a function of the ratio between the gauge-length of sensor and the diameter of the aggregate and (**b**) example of experimental results that show a change in the strain at the location of the sensor due to cracking as a function of the distance between the sensor and the crack tip; the gauge length of the sensors was 5 mm (modified from the slides of author’s university course CEE 537 Structural Health Monitoring); markers in both figures represent measurements and dashed curves represent trendlines.

Short-gauge sensors provide measurements close to the exact (and not average) value of the strain. While this is important to understand local material behavior, it might lack the information at a global structural scale. Having a sensor that can measure the average value of the strain, which considers a material as homogeneous at a macro scale, is thus desirable. The following experimental results show that this is achieved by long-gauge strain sensors. Let us consider the difference between the theoretical estimate of the strain (i.e., average strain) and sensor measurement as the error in strain measurement in concrete, observed at a macro scale. The left graph in Figure 9 shows an example of the experimentally determined dependence of the error on the ratio between the size of the sensor gauge length and aggregate size [8]. The figure shows that indeed, a longer gauge length provides more accurate measurements (note that an excessively long gauge-length of a sensor can also lead to inaccuracy in measurement; however, this is not dependent on material properties but on the strain distribution along the sensor’s gauge length, see [11]).

The second limitation of short-gauge sensors is their relatively small spatial coverage in structure, which reduces the chances to capture the damage. If the damage occurs at the location of the sensor or in its very close proximity, the sensor will detect damage by showing an unusually high change in measured values, as the damage will result in strain field anomaly (see Figure 3a and Figure 9b). This is called “direct detection”, as the sensor is in direct contact with strain field anomaly. However, the damage-induced strain field anomalies have a very localized character. In other words, if the damage occurs even at modest distances from the sensor (typically 25 to 30 mm from sensor), its impact on the strain at the location of the sensor might be negligible, and the damage might pass undetected. To illustrate this statement, the right image in Figure 9 shows results of tests where changes in a measured strain in a steel plate using 5-mm-long strain gauges are plotted with respect to the distance from the point of crack initiation [64]. The figure shows successful detection of damage in very close proximity to sensors, but it also shows that the magnitude of the strain change diminishes rapidly with distance from the damage, in which case the damage might pass undetected. Longer gauge-length improves a sensor’s spatial coverage and thus increases the chances of the sensor being in direct contact with, or in very close proximity to the strain field anomaly, which in turn increases the chances of detecting the damage. Nevertheless, an excessively long gauge length may average the influence of the damage over a length that is too long, which would then result in an insignificant average strain change in the sensing element of the sensor and would consequently reduce the sensor’s sensitivity in damage detection. Thus, the choice of the correct gauge length in a specific project is very important (e.g., see [11]).

The second-generation strain sensors provided not only long-gauge sensors but also truly distributed sensors. Both types of sensors successively addressed the limitations of the first-generation sensors and transformed the way SHM is performed by enabling monitoring at a global structural scale and the integrity scale.

Development of the second generation of strain sensors was enabled by the progress in fiber-optic technologies. In 1870, John Tyndall demonstrated that light can be trapped and guided through a water gush (i.e., thin fiber-like material) in a demonstration to Royal Society. This demonstration is nowadays considered the predecessor of the functioning principle of optical fibers. It took almost a century to create modern silica-based optical fibers as reliable guides of optical signals. Their improvements and widespread use were facilitated and accelerated by developments of lasers in 1960′s, which made possible the control of optical signals and their use in long-distance communication (Theodore Maiman is credited for invention of the first laser in 1960). The experimentation with optical fibers for sensing purposes started almost immediately afterwards.

The physical principles behind the fiber-optic sensors were much more diverse than those behind the first-generation sensors. These principles were mostly discovered in the late XIX and early XX century by famous scientists such as Albert A. Michelson, Lawrence and William Henry Bragg, Ludwig Mach and Ludwig Zehnder, Charles Fabry and Alfred Perot, Rayleigh (John William Strutt), Léon Brillouin, and Chandrasekhara Venkata Raman, many of whom are recipients of prestigious awards including Nobel Prizes. These physical principles resulted in two distinguished groups of sensors: discrete (point) sensors based on Extrinsic Fabry-Perot Interferometry (EFPI), Michelson and Mach-Zehnder Interferometry (so called SOFO sensors), Fiber Bragg-Grating spectrometry (FBG), intensity losses, and (truly) distributed sensors based on Brillouin scattering and Rayleigh scattering (e.g., see [11,65]). Initial exploration in transforming optical fibers into fiber-optic strain sensors (FOSS) started in the 1970′s (e.g., [66,67]) and resulted in the first fiber optic strain sensors and their application in the 1980′s (e.g., [68,69,70,71]). They were rapidly converted into commercial products in the 1990′s, initially by existing companies [72], followed by a surge of startups and new companies.

**Figure 10:**
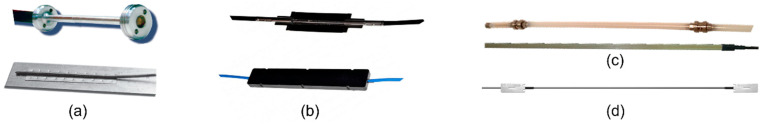
Examples of discrete fiber optic strain sensors: (**a**) short-gauge EFPI sensor, (**b**) short-gauge FBG sensors, (**c**) long-gauge SOFO sensors, and (d) long-gauge intensity-based sensor (photos (**a**–**c**) courtesy of Roctest, Saint-Lambert, QC, Canada, www.roctest.com, last accessed on 28 February 2022 and SMARTEC SA, Manno, Switzerland, www.smartec.ch, last accessed on 28 February 2022; photo (**d**) courtesy of OSMOS Group SA, Paris, France, https://www.osmos-group.com, last accessed on 28 February 2022); sensor sizes in the figure are not to scale.

Discrete fiber optic strain sensors became available as both short-gauge sensors (up to 10 cm in length, e.g., based on FBG and EFPI) and long-gauge sensors (250 mm and longer, up to 2 m, e.g., based on FBGs and intensity, and up to 20 m, e.g., SOFO sensors). Examples of these sensors are shown in Figure 10. The wide choice of sensor types and extensive commercial availability enabled the first widespread SHM applications (with 100+ sensors) at a global structural scale in the 1990′s (e.g., [9,73,74]). The best performances of commercially available discrete fiber-optic strain sensor monitoring systems are given in Table 2.

Availability of long-gauge strain sensors was the first paradigm-changer brought about by fiber-optic strain sensor technologies. They improved accuracy in strain measurements in inhomogeneous materials, improved damage detection capabilities of strain sensors, and enabled monitoring at a global structural scale, where the entire structure could be fitted with sensors, which in turn provided excellent spatial coverage. These improvements provided information on structural behaviors regarding the entirety of the monitored structure (and not only a local part of it) and incited new areas of research in strain-based SHM that were targeting SHM methods and data analysis algorithms (e.g., [9,11,75], etc.).

**Figure 11:**
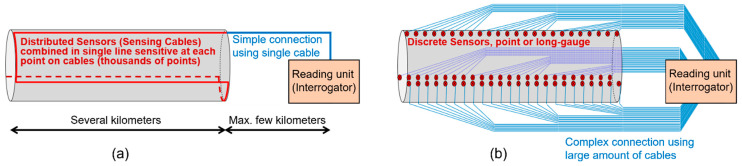
Schematic representation of differences between (**a**) distributed sensing and (**b**) discrete sensing (with parallel multiplexing) of a large structure (modified from the slides of author’s university course CEE 537 Structural Health Monitoring).

The second paradigm-changer brought about by fiber-optic strain sensor technologies is the availability of truly distributed FOSS. A distributed strain sensor is essentially a cable that is sensitive to strain at every point along its length. This disruptive technology practically enabled monitoring of a 1D strain field along the entire length of beam-like structures and provided instrumentation of every cross-section of the structure; in the case of massive or spatial structures, the distributed sensor could be shaped in the form of multiple serpentines to cover large surfaces and volumes. Given that they provide very large spatial coverage, distributed sensors greatly improved the capability of damage detection and enabled integrity monitoring of the entire structure (integrity scale). This capability, in turn, enthused new research related to both distributed sensing and integrity monitoring. Figure 11 schematically shows the advantages of distributed sensing when compared with discrete sensing: (1) a single distributed sensor can replace thousands of discrete sensors, (2) every cross-section of the structure is instrumented with a distributed sensor, which is not the case with discrete sensors (i.e., there are “gaps” between discrete sensors), and (3) in distributed sensing, connection to reading unit is significantly simplified, which reduces the cost of installation and maintenance.

**Figure 12:**
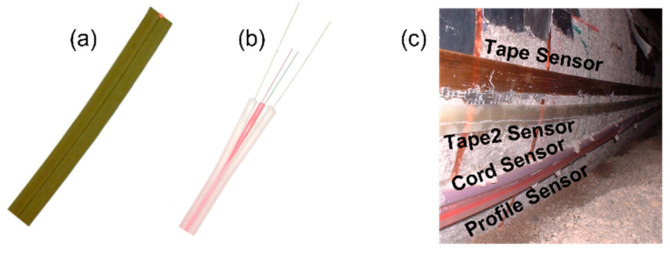
Examples of distributed fiber optic strain sensors: (**a**) tape sensor and (**b**) profile sensor (photos courtesy of SMARTEC SA, Manno, Switzerland, www.smartec.ch, last accessed on 28 February 2022), and (**c**) different types of distributed sensors installed on a pipeline specimen (modified from the slides of author’s university course CEE 537 Structural Health Monitoring); sensor sizes in the figure are not to scale.

Research on distributed FOSS started approximately one decade after the start of the research on discrete FOSS. First, distributed temperature sensing was discovered in the 1980′s (e.g., [76,77,78]), shortly followed by strain sensing (e.g., [79]). A breakthrough in distributed strain sensing was made in the 1990′s, with the development of sensing techniques based on Brillouin and Rayleigh scattering. Similar to earlier research, Brillouin and Rayleigh effects were first studied in the context of temperature sensing (e.g., [80,81,82,83]), followed by strain sensing (e.g., [84,85,86,87]). Distributed sensing reached the market at the end of the XX and early XXI centuries. Several new companies were created, but they mostly focused on manufacturing reading units based on proprietary technology. The commercialization of distributed sensors, specifically designed for strain monitoring, followed soon thereafter (e.g., [88,89]) and led to the first large-scale applications at a global, integrity scale (e.g., [33,90,91,92]). Examples of distributed sensors are shown in Figure 12.

While FOSS are, at the present day, mature, well-established, and widely accepted tools for SHM, they still represent a vibrant area of research, which targets improvement of their performances. Examples of research topics are new sensors for high-temperature environments [92,93], a Brillouin-based sensing system for dynamic monitoring [34,94], improved spatial resolution of Brillion-based sensing systems (in a range of several mm to several cm) [37,95], measurement of strain and temperature using single fibers [96], new applications for distributed FOSS [35,97,98,99,100], etc. The best performances of commercially available distributed fiber-optic strain sensor monitoring systems are given in Table 3.

While each specific type of fiber-optic strain sensor has different advantages and limitations, depending on physical principle and components, the second-generation strain sensors have some common advantages and limitations, as listed below.

General advantages:

- Long-term stability and durability: optical fibers were originally designed for the purposes of telecom industry, and thus, they are designed to be chemically inert, with stable material properties in the long term (they were extensively tested to prove these properties); the main component of FOSS is a standard optical fiber, the same as the one used in the telecom industry, which provides long-term stability and durability to FOSS.
- Electrical passivity and reliability: FOSS are electrically passive and thus they are not affected by electro-magnetic interference; this property along with long-term stability enables long-term reliability of sensor measurements; moreover, electrical passivity makes FOSS applicable in the environments where the use of electrical sensors is forbidden due to potential sparking (e.g., in the gas and oil industry).
- Excellent measurement performance: the second-generation strain sensors feature high resolution, typically in the range of 0.2–5 με, and precision typically ranges between 1 με (for discrete FOSS and Rayleigh-based distributed FOSS) and 20 με (Brillouin-based distributed FOSS); these measurement performances are suitable for the SHM of civil structures.

General challenges:

- Cost: one quarter of a century after reaching market maturity, the cost of the second-generation sensors is still on average higher than the cost of the first-generation sensors, which is due to use of expensive components and manufacturing processes; however, it is important to note that the cost of FOSS did not increase over time and thus, the difference in cost between the second- and the first-generation sensors has steadily decreased and is expected to further decrease in the near future.
- Tedious repair of cable extensions in the case of damage: being made of optical fibers, the cable extensions are not easy to repair and replace onsite: connection (splicing) of optical fibers requires special equipment that might not be easy to handle and operate under on-site conditions.
- Wireless capability: the FOSS cannot be interfaced wireless nodes, as they operate using optical signal; thus, the FOSS must be wired to the reading unit or channel switch; however, the fiber-optic strain sensor reading unit has the capability of wireless communication with the remote office location.

More details regarding the history and performance of the second-generation sensors can be found in relevant literature (e.g., [11,65,95,101,102]).

## 4. Third Generation: Distributed and Quasi-Distributed Two-Dimensional (2D) Strain Sensors

Strain sensors from the first two generations responded well to the first two needs mentioned in the introduction of this paper, i.e., to help assess stresses and deflections as indicators of safety and performance. However, damage characterization, while successfully addressed in many cases, remains very challenging in general. There are two approaches in damage detection based on strain sensing: direct and indirect approaches.

The indirect approach assumes that sensors are not placed at locations where the damage occurs. In this case, the damage may or may not change the strain field at locations where the sensors are installed. If the damage results in the change in strain at locations of sensors, that change can be used as an indicator of damage. However, the challenges with this approach are numerous. First, as mentioned earlier, the strain field at locations even a modest distance from the damage is only lightly perturbed, and this perturbation is difficult to discriminate from usual noise caused by changes in loads and environmental conditions (e.g., [64,103]). Second, given that the detection of damage in that case cannot rely on strain change only, advanced data analysis algorithms have to be created and used to ascertain the existence of damage; nonetheless, implementation of these algorithms can be complex and expensive, without guarantee in the success in real-life settings. Finally, in some situations, no algorithm can be used in combination with strain sensors to indirectly ascertain the damage: for example, damage in statically determined structures will not cause any redistribution of strain at locations that are even slightly distant from the damage origin. Thus, the indirect damage detection approach, while successfully implemented in many applications, has limitations that cannot be overcome in general.

The direct sensing approach assumes that the sensor is placed at the location of damage occurrence; damage would create strain field anomaly, which is, in turn, simply detected by the sensor as an unusually high change in the measured strain (e.g., see Figure 3a). The advantage of this approach is very high reliability in damage characterization, which is a result of correct placement of the sensor and the changes in strain measurements caused by the anomaly (a simple threshold can be used to detect the damage, e.g., [11,27,28,31,64]). However, the challenge in this approach is that very frequently the location of damage occurrence cannot be accurately predicted. This challenge can be mitigated by deploying large numbers of densely spaced discrete sensors, but this would be very expensive in terms of both the cost of the equipment and the cost of installation. 1D Distributed sensors would be a better choice as they can cover larger areas using single sensor, and they have been successfully applied for direct damage detection in many projects (e.g., [27,90]). Nevertheless, they also have limitations that are imposed by the geometrical properties of the monitored structural members in terms of the 2D surface, texture, angles, and size. Hence, there is a need for new type of sensor that can enable direct damage detection and characterization, in general cases.

The research targeting solutions for affordable direct sensing resulted in the creation of the third generation of strain sensors. The research approximately began at the turn of the XXI century and is currently in the prototyping phase, with some first applications on real structures being realized during the second decade of the XXI century. As opposed to the first two generations of strain sensors where development was mostly technology-specific and oriented towards sensors with high strain measurement performances, the third generation was chiefly (but not only) aimed at sensors that can be deployed over large areas of structures and that use strain change as a damage indicator. Thus, the emphasis was on large spatial coverage at the expense of accuracy in strain measurement. This was justified as the sensors for accurate strain measurement already exist (the first- and the second-generation sensors) and, to complement SHM solutions, damage can be directly detected with less accurate sensors of the third generation. For example, when cracks open over the sensor, these generate strain measurement that is several orders of magnitude higher than the usual strain change due to the load and environmental effects; such a large change can be detected with sensors that are an order of magnitude less accurate than sensors of the first and the second generation. The active research on third-generation strain sensors deals with two general approaches: contact and non-contact sensing.

**Figure 13:**
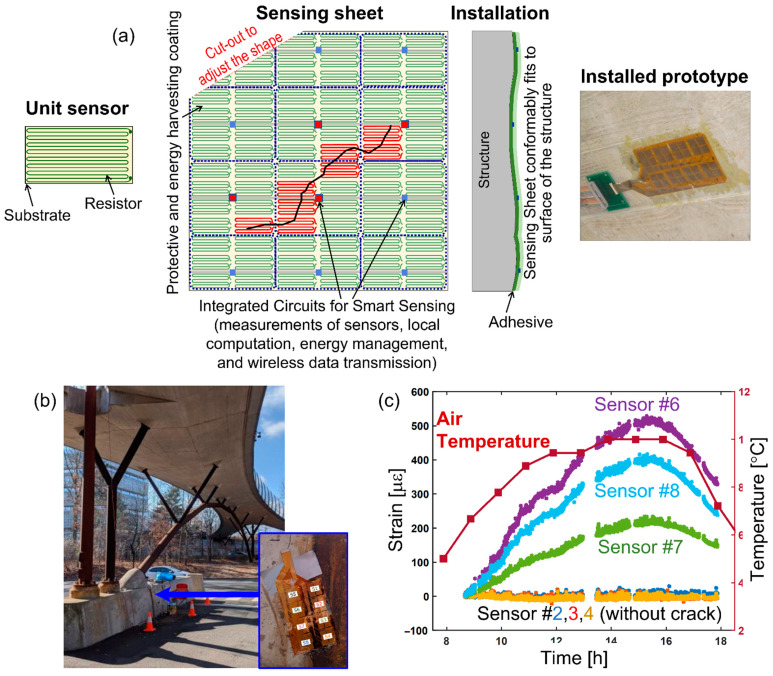
Example of a quasi-distributed 2D sensing sheet: (**a**) schematic of sensor components (modified from the slides of author’s university course CEE 537 Structural Health Monitoring), (**b**) prototype installed over a shrinkage crack on Streicker Bridge foundation (modified from [118]), and (**c**) results of measurements showing the crack opening over time (modified from [118]).

The research on contact sensing approaches was the first to start. It yielded prototypes in various forms of quasi-distributed sensors such as expandable sensor networks (e.g., [104,105]) and sensing sheets (e.g., [64,106,107]), truly distributed sensors, such as photonic crystals (e.g., [108,109]), sensing skins (e.g., [110,111,112,113]), and nano-paints and nano-based materials and adhesives (e.g., [114,115,116,117]), etc. As an example, a schematic of a 2D sensing sheet is shown in Figure 13, along with its application on a bridge and its preliminary assessment of cracking damage.

**Figure 14:**
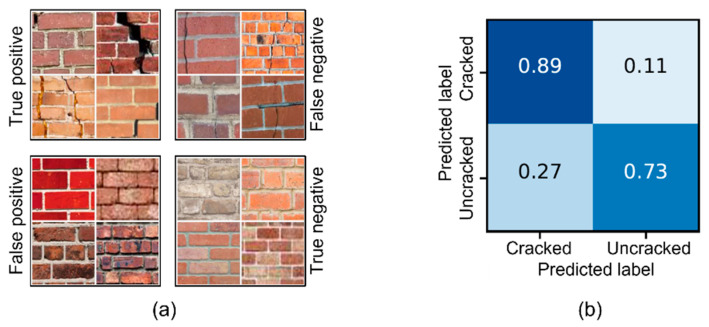
(**a**) Examples of imaging (randomly selected) and (**b**) confusion matrix for the crack detection method using digital image processing based on convolutional neural networks [121].

The research on non-contact techniques started in the second decade of the XXI century and is mostly based on digital image processing approaches. Generally, digital images of structural areas can be repeatedly taken over time and compared to each other or to a reference image using specialized algorithms. Digital image processing at a very fine (pixel) scale can be performed using a combination of powerful processors and machine learning, which results in mapping of the displacement of points (pixels). The displacement map can be then used to indirectly evaluate the strain field of the photographed structural area (e.g., [119]) or to identify unusual structural behaviors (e.g., damage [120]). In addition, digital image processing can be used to directly identify visible damage, such as cracking or spalling (e.g., [121,122]). Examples of images used in crack detection based on convolutional neural networks are shown in Figure 14, along with a confusion matrix that indicates the performance of the method.

The great advantage of the third-generation sensors is their potential to detect and characterize damage over 2D structural surfaces by direct sensing, which can significantly improve the reliability of damage identification in a real-life setting, i.e., under varying environmental conditions. This, in turn, has potential to open the doors for 2D integrity monitoring at a large scale.

The main challenge of third-generation sensors is scaling to the large size of structures, especially concerning their deployment (e.g., bonding over large areas of structures or photographing large areas of structures), long-term reliability, and data analysis and management. Nevertheless, many of the third-generation sensors will certainly reach the market and become standard tools for the assessment of real structures.

Besides the above-presented 2D distributed and quasi-distributed sensors, the research on third generation sensors also includes discrete sensors based on micro-electro-mechanical systems (MEMS). Sensors based on MEMS have been researched since the 1990′s (e.g., [123]), and were employed in mechanical engineering and biomedical applications, mostly as sensing elements for other types of sensors (e.g., force sensors). However, although their applicability in the strain-based SHM of civil structures is not yet fully proven, very promising research is ongoing (e.g., [124,125]).

## 5. Conclusions and Future Research Directions

**Figure 15:**
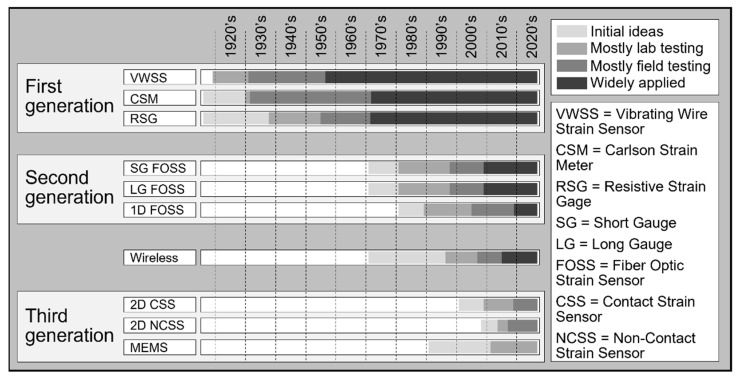
Condensed timeline of the development of strain sensors and strain-based sensing techniques used in the SHM of civil structures (modified from the slides of author’s university course CEE 537 Structural Health Monitoring).

**Figure 16:**
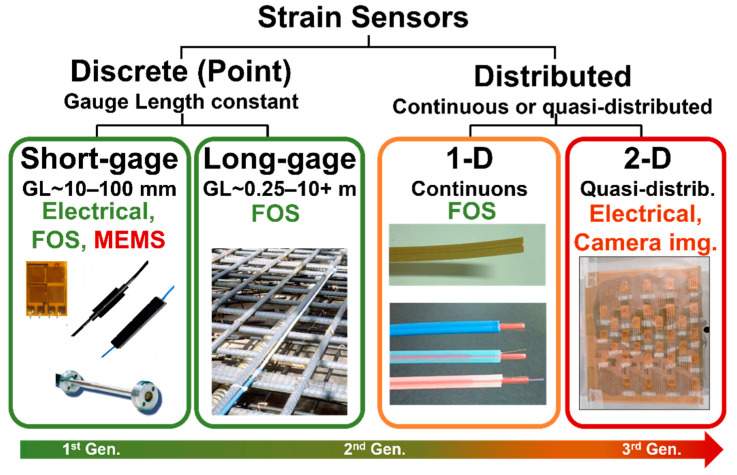
Progress in spatial coverage (gauge length) of strain sensors (modified from the slides of author’s university course CEE 537 Structural Health Monitoring); color-coding: green = mature; orange = mature in part, but research is still needed; red = under research and development (photos of FOSS in the image are courtesy of SMARTEC SA, Manno, Switzerland, www.smartec.ch, last accessed on 28 February 2022).

Strain sensors have been developed and used for the SHM of civil structures for more than one hundred years. This period is characterized by three generations of sensors that pushed boundaries and significantly expanded the capabilities of strain-based SHM. A chronology of their development is shown in Figure 15. Generational progress in their spatial coverage is shown in Figure 16.

The first-generation sensors consist of discrete (point) short-gauge sensors based on electrical principles. Both surface-mounted and embeddable variants were developed. They enabled elementary evaluation of strain at a local material level and opened the doors for strain-based SHM.

The second-generation sensors consist of discrete short-gauge and long-gauge sensors, and 1D distributed sensors, all based on fiber optic technologies. Short-gauge sensors brought about properties that were complementary to the first-generation sensors. However, the real paradigm-changers were long-gauge and 1D distributed sensors, as they enabled the monitoring of materials at a macro-scale, large spatial coverage of structures, and improved damage detection capabilities, thus enlarging the scale of SHM to global and integrity monitoring. These paradigm changes and their generational progress are illustrated in Figure 16.

**Figure 17:**
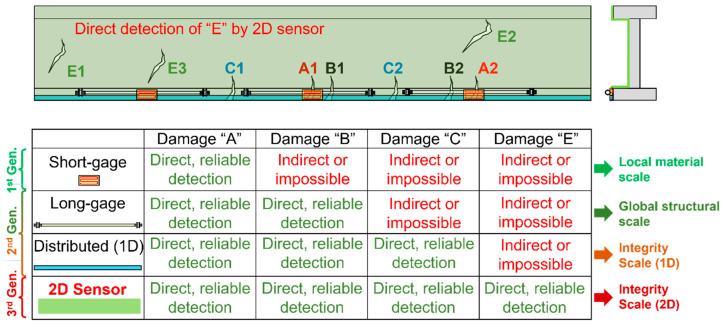
Progress in damage detection capabilities of strain sensors and their transformative impact on the scale of applicability in SHM (modified from the slides of author’s university course CEE 537 Structural Health Monitoring).

The third generation of strain sensors addresses the needs for even larger spatial coverage, greater spatial resolution, and improved reliability in damage detection. Currently, the third-generation sensors include a large variety of technologies and focus mostly (but not only) on truly distributed (continuous) or quasi-distributed 2D strain sensors. Two main categories of these sensors are contact and non-contact sensors. The paradigm change they are bringing about is the shift from accurate strain measurement to direct sensing of damage, i.e., the use of the strain as a damage indicator rather than the main observed parameter. Figure 17 shows generational progress in capabilities in performing damage detection by direct sensing and their transformative impact on the scale of applicability in SHM.

It is important to highlight that progress in strain-based SHM could not be possible without convergent research that included achievements in other branches of science and engineering. Important milestones were developments in communication technologies, electrical and computer engineering, and mathematics and computer science, which resulted in the creation of wireless technologies that greatly improved remote SHM, the development of statistics, and machine learning techniques that enabled new approaches in data analysis, which, in turn significantly improved the understanding of the data collected by sensors, and the creation of powerful processors and servers that enabled fast computation, access to, and storage of big data.

Current and future research and development in strain sensing have various directions. One, that is already being carried out, mentioned earlier in the text, addresses the need for improved properties of 1D-distributed FOSS, in particular to enable dynamic monitoring. Another one, also being carried out, regards the completion of research and development of 2D distributed and quasi-distributed sensors, so they can reach market maturity.

An important research direction that is also currently being explored is full understanding of data collected by currently existing strain sensing techniques and the assessment of capabilities of strain measurements to be used for the prediction of future structural behaviors using data-driven approaches (statistics and machine learning, e.g., [126,127]). While this direction does not directly address the development of sensors, it is extremely important as it emphasizes the importance of sensors beyond simple strain measurement and has the potential of enabling smart structures and transforming them into cyber-physical systems (e.g., [128]).

Future research certainly should address the challenge of affordable 3D distributed and quasi-distributed strain sensing so that the entire structure or structural component can be monitored at all three scales, local, global, and integrity scales. For example, based on promising preliminary results, good candidates for such an exploit are sensors based on radiofrequency back-scattering (e.g., [129]). Another future direction could be exploration of digital approaches that can enable human-structure interactions in strain measurement, such as virtual and augmented reality, where preliminary results are very promising (e.g., [130]).

An important challenge that the strain sensors of all generations face is long-term stability and reliability. While some of the sensors of the first two generations have proven their excellent long-term stability and reliability over a few decades, many have shown a drift or malfunction due to ageing of sensor components caused by various environmental factors (chemical agents, electromagnetic interferences, temperature fluctuations, etc.). Given that structures are designed to serve for several decades, it will be important for sensors to match their longevity. Thus, an important challenge that should be addressed in the future is the longevity of sensors of all generations—current and forthcoming.

Finally, one should not forget to be vigilant regarding discoveries in other areas of science and engineering, as they will certainly provide new technologies that can be used for future generations of strain sensors. Only convergent research in collaboration with other disciplines can result in new paradigm changes in strain-based SHM.

In conclusion, one century after the development of the first strain sensors and almost one century after their first real-life applications, strain sensors based on several technologies are, at present day, commercially mature and are widely and successfully applied in many real-life SHM projects. They have enabled better understanding, optimized maintenance, and improved safety of civil structures worldwide. They gave birth to many companies and an entire industry sector. Regardless of their long tradition, strain sensors and strain-based SHM still represent vivid areas of research, development, and innovation. 

## Figures and Tables

**Table 1:** Best performances of the commercially available first-generation (electrical) strain sensors used in civil SHM applications (best performances, often, cannot be achieved simultaneously).

Property	Vibrating Wire Strain Sensor	Resistive Strain Gauge	Carlson Strain Meter
Gauge length	Typical: 50–150 mm Special (long): 300 mm	Typical: 0.3–20 mm Special (long): 150 mm	Typical: 203–508 mm Special (short): 102 mm
Multiplexing (the way multiple sensors are accessed by the reading unit)	Parallel (one by one)	Parallel (one by one)	Parallel (one by one)
Maximum number of sensors per reading unit (with a channel switch or multiplexer)	32 *	1200 *	24 *
Stability	Long-term stable	Long-term drift (typical)	Long-term stable
Resolution	Typical: 1 με Best: 0.35 με	Typical: 1 με Best: 0.5 με	Typical: 2–3 με Best: 1.5 με
Linearity (repeatability precision)	Typical: ±0.5% Full Scale Best: ±0.1% Full Scale	0.2–2%	N/A
Sensor range	Typical: 3000 με Extended: 10,000 με	Typical: ±10,000 με Extended: ±100,000 με	Typical: 2000 με Extended: 3900 με
Temperature sensitivity	Compensated with an integrated temperature sensor	Compensation needed	Self-compensated (by measurement principle)
Measurement frequency	Typical: 0.25–1 Hz Dynamic: 20 to 333 Hz Max.: 1 kHz *	Typical: 100–200 Hz Max. 100 kHz	N/A

* Unconfirmed.

**Table 2:** Best performances of the commercially available second-generation (fiber-optic) discrete strain sensors used in civil SHM applications (best performances, often, cannot be achieved simultaneously).

Property	Extrinsic Interferometry (EFPI)	Intrinsic Interferometry (SOFO)	Fiber Bragg Gratings (FBG)	Intensity Based (Micro-Bending)
Gauge length	51 to 70 mm	250 mm to 20 m	10 mm to 2 m	100 mm to 10 m
Multiplexing	Parallel	Parallel	In-line and parallel	Parallel
Max. number of sensors per reading unit	32	Static: unlimited Dynamic: 8	80 to 300 (depending on reading unit)	80
Stability	Long-term stable	Static: long-term stable Dynamic: short-term stable	Long-term stable	Long-term drift (typical 2%)
Resolution	0.01% full scale	Static: 2 μm/gauge length Dynamic: 10 nm/gauge length	0.2 με	1 μm/gauge length
Repeatability (precision)	N/A *	Static: 0.2% full scale Dynamic: N/A *	1 με	1%
Sensor range	±3000 με	Static: −5000 με to +10,000 με Dynamic: ±5 mm/gauge length	−5000 με to +7500 με	±5000 με
Temperature sensitivity	Insensitive (by measurement principle, but might be packaging dependent)	Self-compensated (by measurement principle)	Compensation needed	0.6 μm/°C
Measurement frequency	20 Hz	Static: 0.1 Hz Dynamic: 10 kHz	0.5 MHz	100 Hz

* Unconfirmed.

**Table 3:** Best performances of the commercially available second-generation (fiber-optic) distributed strain sensors used in civil SHM applications (best performances, in general, cannot be achieved simultaneously).

Property	Stimulated Brillouin Scattering	Spontaneous Brillouin Scattering	Rayleigh Scattering
Spatial resolution (equivalent to gauge length)	0.2 to 5 m	1 m	10 mm
Sampling interval (min. space between measurement points on sensor)	100 mm	50 mm	0.65 mm
Maximum number of sensors per reading unit (with channel switch or multiplexer)	16	N/A	8
Stability	Long-term stable	Long-term stable	Long-term stable
Resolution	2 με	2 με	0.1 με
Reproducibility (“accuracy”)	±2 to ±50 με	±20 με	±30 με
Sensor range	±10,000 με	±10,000 με	±15,000 με
Max. sensor length	50 km	25 km	100 m
Temperature sensitivity	Compensation needed	Compensation needed	Compensation needed
Measurement frequency	10 sec. to 15 min.	4 to 25 min.	250 Hz.
